# Seed-to-plant-tracking: automated phenotyping of seeds and corresponding plants of Arabidopsis

**DOI:** 10.3389/fpls.2025.1539424

**Published:** 2025-04-28

**Authors:** Daniel Klasen, Andreas Fischbach, Viktor Sydoruk, Johannes Kochs, Jonas Bühler, Robert Koller, Gregor Huber

**Affiliations:** Institute of Bio- and Geosciences: Plant Sciences (IBG-2), Forschungszentrum Jülich GmbH, Jülich, Germany

**Keywords:** automated phenotyping, *Arabidopsis thaliana*, seed, early vigor, germination, trait distribution, seed morphology, heat treatment

## Abstract

Plants adapt seed traits in response to different environmental triggers, supporting the survival of the next generation. To elucidate the mechanistic understanding of such adaptations it is important to characterize the distributions of seed traits by phenotyping seeds on an individual scale and to correlate these traits with corresponding plant properties. Here we introduce a seed-to-plant-tracking pipeline which enables automated handling and high precision phenotyping of Arabidopsis seeds as well as germination detection and early growth quantification of emerging plants. It includes previously published measurement platforms (*pheno*Seeder, Growscreen), which were improved for very small seeds. We demonstrate the performance of the pipeline by comparing seeds from two consecutive generations of elevated temperature during flowering with control seeds. Relative standard deviation of repeated seed mass measurements was reduced to 0.2%. We identified an increase in seed mass, volume, length, width, height, and germination time as well as a darkening of the seeds under the treatment. A correlation analysis revealed relationships between seed and plant traits, e.g., a highly significant negative correlation between seed brightness and germination time, and a positive correlation between seed mass and early growth rate, but no correlation between time of emergence and morphometric seed traits (e.g., mass, volume). Thus, the seed-to-plant tracking provides the basis for investigating the mechanism of seed and plant trait variation and transgenerational inheritance.

## 1 Introduction

Seeds are not only the starting point of every new emerging plant, but in many cases also the product of agriculture (Wang and Smith, 2002; Mattana et al., 2021). Therefore, seed traits are economically important as well as of scientific interest in the context of inheritance and adaptation of plants to changing environments. Some seed traits are easy to obtain. For instance, thousand kernel weight, which is frequently employed as an indicator for yield and, consequently, for the performance of a crop (Kulp and Ponte, 2000). While this is a common approach it does not consider variability inside the seed batch. A wide distribution of seed traits can be beneficial for survival of a species. In non-optimal growth conditions, the mother plant is capable of reducing uniformity in germination of the next generation. This so called bet-hedging strategy helps to minimize the risk of overall germination failure (Cohen, 1966). In spite of this general importance, it is still not fully understood whether, or to what extent, the variability of seed traits (e.g. volume, mass and colour) within plant species or genotypes has an impact on seed emergence (i.e. germination), early development and further performance of a plant (Joosen et al., 2012; Krzyszton et al., 2024). Distributions of seed traits are typically rather wide (Jahnke et al., 2016). Therefore, the characterization of variability in seed traits requires measurement of a large number of individual seeds. This can be difficult especially with very small seeds such as those of the model plant Arabidopsis (*Arabidopsis thaliana*) (Somerville and Koornneef, 2002). Here, handling of seeds as well as precision and reproducibility of trait determination poses challenges (Jahnke et al., 2016; Dani and Kodandaramaiah, 2017) and calls for automated phenotyping procedures.

Morales et al. (2020) used a large-particle flow cytometer for high-throughput sorting of Arabidopsis seeds. The measured seed trait was individual projected seed area, similar to a scanning procedure. The seeds were sorted and stored in batches, so that it is not possible to track specific seeds and their emerging plant. In the Boxeed^©^ platform for handling individual seeds (Krzyszton et al., 2024), the projected seed area is retrieved and the seeds are placed in a specific position on agar. This enables the combination of seed and germination properties. Limitations are the absence of volumetric seed trait measurement as well as the missing option to continue the plant life cycle until harvest. The robotic system *pheno*Seeder (Jahnke et al., 2016) automatically handles individual seeds of different sizes, measures morphometric traits (such as seed mass, volume, length, width, and height) and can be used for sorting or sowing. Seed volume is calculated from optical measurements by volume carving (Roussel et al., 2016). Seed mass is obtained using different dedicated balances depending on the seed size. The *pheno*Seeder is taking RGB images which can be used to quantify the optical appearance of a seed coat (testa). This is of interest because there is evidence that the testa influences dormancy as well as seed longevity (Debeaujon et al., 2000). Limitations of the *pheno*Seeder are handling issues and accuracy for very small seeds such as Arabidopsis and a restricted throughput. Sowing is typically done directly in soil. This enables the possibility to follow the plants till the end of their life cycle.

In addition to the mentioned automated seed phenotyping systems, several approaches were developed to automate measurement of Arabidopsis seedling growth (Walter et al., 2007; Apelt et al., 2015; Demidchik et al., 2020; Samiei et al., 2020). The working principle is typically simple and consists of the following steps: an optical sensor determines the rosette of Arabidopsis plants, from this the leaf area is estimated and taken as proxy of plant biomass. Growth rates are calculated from time series of leaf areas.

**Figure 1:**
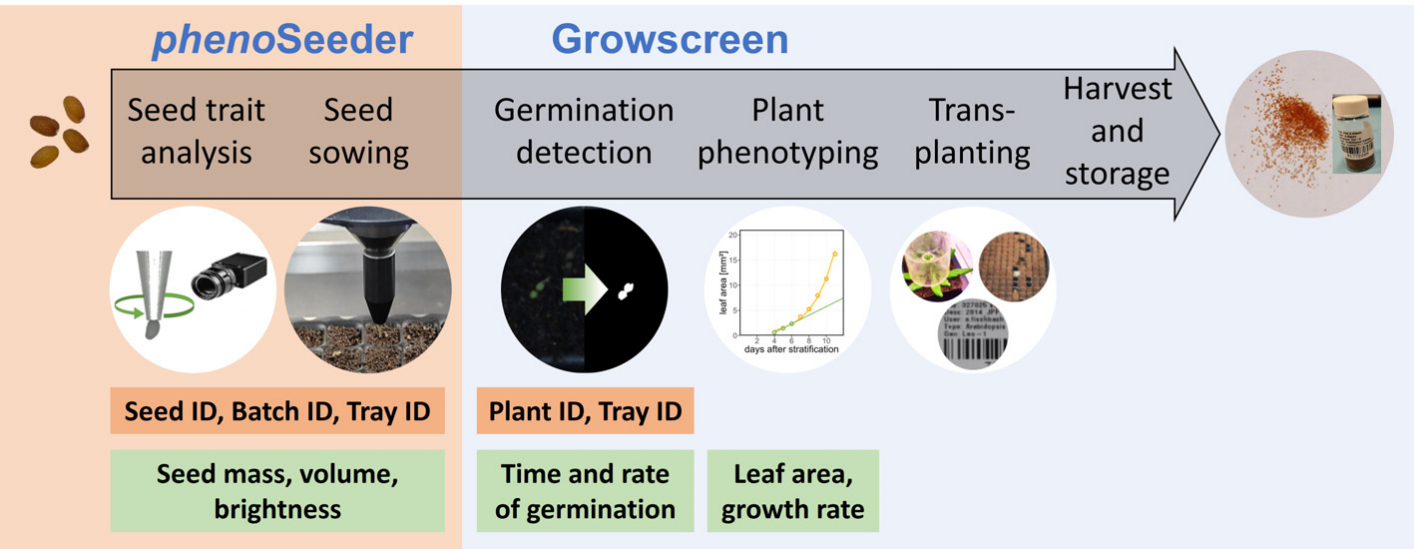
Schematic of seed-to-plant tracking. Main modalities are the phenoSeeder for automated determination of seed traits and seed handling; the Growscreen for germination detection and plant phenotyping. IDs assigned for tracking seeds and plants are listed in orange boxes. Green boxes display traits retrieved from measurements.

In this study, we present a seed-to-plant tracking pipeline (**Figure 1**) for automatically phenotyping individual seeds and corresponding plants of Arabidopsis. Within this pipeline seeds are phenotyped using the *pheno*Seeder, sown into soil, and the emergence and early development of plants is characterized by the Growscreen system (Walter et al., 2007). We describe the improvements made to the *pheno*Seeder system to enable a precise automated phenotyping and handling of the very small seeds of Arabidopsis. We demonstrate the capabilities of the pipeline for seeds of Arabidopsis Columbia-0 grown under different temperature regimes. We focus on using the phenotyping data to investigate correlations between seed traits and early growth of corresponding seedlings.

## 2 Materials and methods

### 2.1 Plant growth conditions and seed production

*Arabidopsis thaliana* Columbia-0 (Col-0) seeds were sown in seedling trays with 32 x 18 pots of 1.5 x 1.5 cm, filled with soil “Einheitserde Typ Pikier” (Balster Einheitserdewerk GmbH, Fröndenberg, Germany). After sowing the seeds were stratified for at least 48 hours at 4°C in dark humid conditions. After stratification the trays were placed in a climate chamber. The photoperiod was set to 16 h at a stable temperature of 20°C and 50% humidity. Light intensity ranged between 160 to 180 µmol/(m²s). Plants were positioned in the centre of the shelves to minimize any effect of light inhomogeneity on plant growth. Additionally, the position of the trays was randomized daily.

After 14 days, randomly selected Col-0 plants with at least two true leaves were transplanted on the same day into 7 x 7 cm pots filled with “Lignostrat Dachgarten extensive” soil (Hawita Group GmbH, Vechta, Germany) for further growth under the same conditions as before transplantation. Plants were continuously observed for flowering. Flowering was defined as shoot larger than 5 cm and appearance of the first 4 flowers. All plants reaching those requirements were contained using an Aracon (BETATECH BVBA, Ghent, Belgium). Half of the flowering plants were transferred into elevated temperature (26°C). During the further growth the plants were trimmed when starting to grow outside of the Aracon. 12 weeks after stratification the watering was stopped, and plants were left to dry for two weeks. All plants per treatment were harvested together and the seeds were pooled in single batches. Seeds were cleaned by sieving them (approx. 1 mm mesh size) multiple times and stored in a 20 ml glass vial (PerkinElmer LAS GmbH, Rodgau, Germany) at 20°C for at least 4 weeks till phenotyping was conducted.

The whole protocol from sowing to harvest was followed over two consecutive generations. In each generation, 72 out of 100 phenotyped seeds per batch were sown in soil, 20 emerged plants per batch were transplanted, out of which 10 plants were exposed to elevated temperature, whereas the other 10 plants remained under control conditions. This procedure resulted in several seed batches, from which the following were chosen for this study: the starting material (‘G0’), a control batch with no treatment over the two generations (‘C’), and a treatment batch (‘T’) with two generations of elevated temperature. For the correlation analysis of seed and plants traits in section 3.2, seeds of the C and T batches were phenotyped and sown again according to the protocol described above, but not transplanted anymore.

### 2.2 Seed-to-plant tracking pipeline

Our pipeline of seed-to-plant tracking for Arabidopsis primarily involves the phenotyping platforms *pheno*Seeder (Jahnke et al., 2016) and Growscreen (Walter et al., 2007; Scharr et al., 2020) as well as a database for data storage (Scharr et al., 2020) (**Figure 1**). Briefly, every seed batch is assigned to a batch ID which is linked to the experiment and the person responsible, the genotype and if necessary, the treatment. For investigation of the relation between seed traits and plant traits each seed is assigned a seed ID when it is picked up by the *pheno*Seeder. The seed ID is linked to the corresponding batch ID and the time point of measurement. After determination of seed traits (such as seed mass, seed volume, colour, projected area, length, width and height), the seed is typically delivered to a defined position on a tray. Tray ID and the x- and y-positions on the tray are stored and linked to the seed ID. In accordance with Scharr et al. (2020) we define germination as emergence of a seedling optically detectable by the Growscreen, i.e., reaching of a threshold of 10 green pixels, corresponding to 0.013 mm². For seedlings with successful emergence, the time point of germination as well as growth traits (i.e., 2D leaf area) of the plant are recorded.

For transplantation into bigger pots and further growth, plants can be selected by germination time, projected leaf area or randomly. Every pot gets a barcode with a newly created plant ID corresponding to the seed ID. This is important for further evaluation, e.g., characterization of plant developmental stages and timing of sampling or harvest. Finally, if plants are designated for propagation, a new batch ID can be generated for each harvested plant, closing the cycle and enabling trait tracking over generations.

### 2.3 *pheno*Seeder amendments for very small seeds

**Figure 2:**
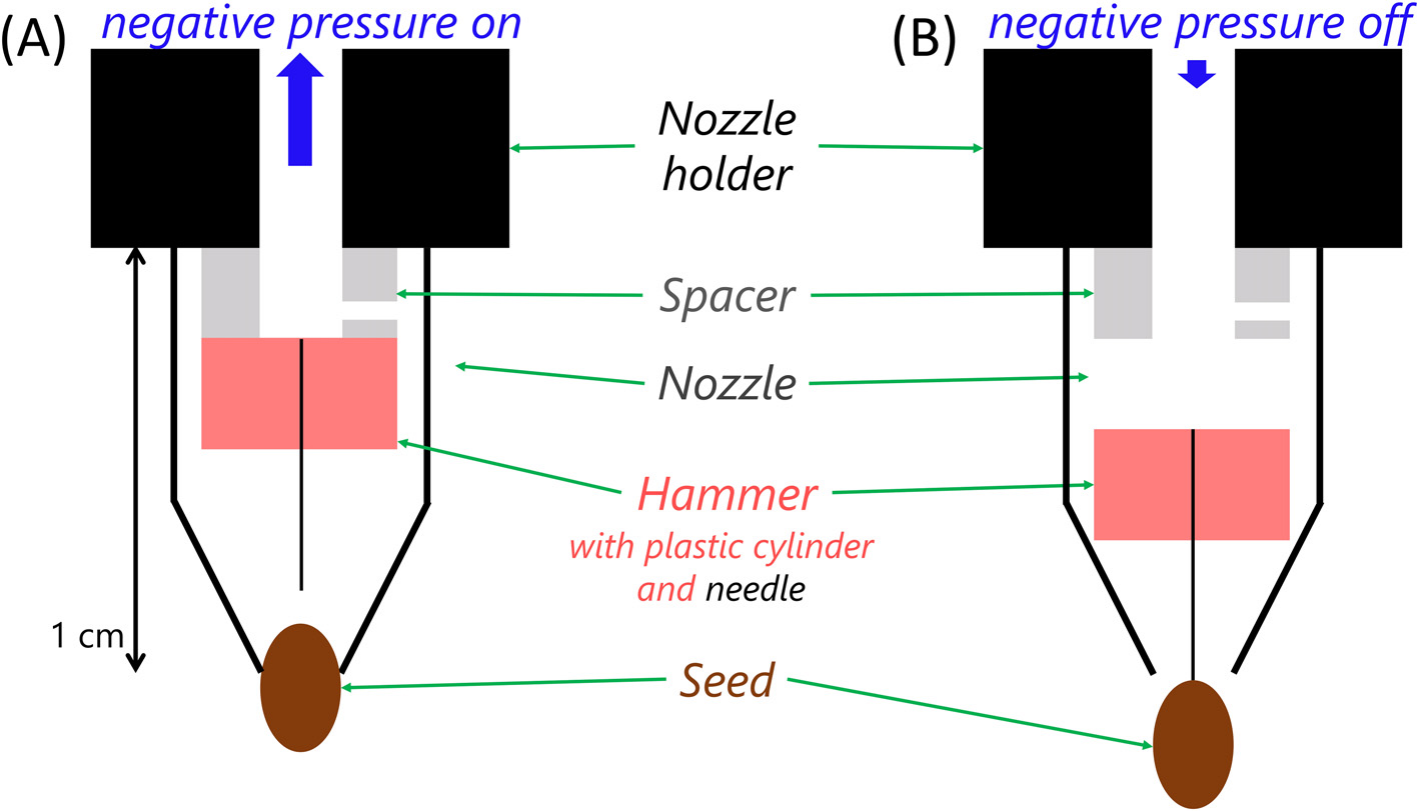
Schematic of new ‘hammer’ release system as replacement of the pneumatic release of Jahnke et al. (2016). **(A)** Under negative pressure the ‘hammer’ is sucked up inside the nozzle and the seed is picked up; **(B)** if the negative pressure is turned off, the ‘hammer’ falls by gravity and releases the seed. The spacer controls the height from which the hammer falls.

The setup of the *pheno*Seeder is explained in detail in Jahnke et al. (2016). Very briefly, in a 2D imaging station single seeds from a seed batch laid out on a glass plate are identified, and seed images are segmented from the background for determination of colour and projected area. After picking up a seed from the 2D station, the robot moves it to a 3D imaging station, where it is rotated in front of a camera (as depicted in **Figure 1**) taking 36 images at 10° steps. From these images seed volume, length, width, and height are estimated (see **Supplementary Data Sheet 1**). Afterwards, the seed is weighed on a dedicated balance (see below) and either sown directly or moved to a storage plate. In the following, we elaborate on the amendments introduced to the *pheno*Seeder in order to achieve a higher precision and a more reliable seed handling for very small seeds. For the latter, the handling tool of the *pheno*Seeder was redesigned. In the previous version of the *pheno*Seeder, seeds were pneumatically picked up by negative and released by positive pressure. For very small seeds, the overpressure during release causes a high acceleration of the seed in the fast air flow: e.g., with an overpressure of *p* = 30 mbar and an inner nozzle radius of *r* = 75 µm, an Arabidopsis seed of mass *m* = 20 µg could receive an initial acceleration of up to *a* = *πr*²*p*/*m* = 2650 m/s², thus reaching a speed of up to *v* = (2*ad*) ^1/2^ = 5.1 m/s over a distance of *d* = 5 mm from the nozzle. This high speed might lead to a faulty positioning of the seed due to bouncing, especially if the seed is released on top of a hard surface. To prevent such issues a new releasing system was introduced with a mechanical device for release instead of a high overpressure. This so called “hammer” is placed in the nozzle and consists of a needle and a cylindrical plastic weight. Under negative pressure, the hammer as well as the target seed are sucked up (**Figure 2A**). After switching off the negative pressure, the hammer falls due to gravity and application of a very small overpressure. The needle slides through the hole of the nozzle, thus releasing the seed (**Figure 2B**) and closing the hole to prevent further air flow out of the nozzle. We adjusted the falling height of the hammer to about 1 mm. Due to the large difference in mass between the hammer (7.2 mg) and the seed (approx. 20 µg), the seed might reach twice the speed of the hammer, which is only up to about 0.2 m/s, supposing totally elastic collision and no energy losses during the fall of the hammer. The lower speed of the seed leads to a more reliable placement, which ensures a positioning of the seeds in the centre of the pots, prevents weighing failures caused by a seed bouncing out of the seed receiver, and enables multiple releases and uptakes for repeated measurements of the same seed (section 3.1).

As already pointed out in Jahnke et al. (2016), the previous *pheno*Seeder balance with a resolution of 1 µg was not sufficient for a precise characterization of individual Arabidopsis seeds. Therefore, it was replaced by a new balance with a resolution of 0.1 µg (MSA 2.7S-CE, Sartorius GmbH, Germany).

The method to estimate seed volume using 3D surface reconstruction (Roussel et al., 2016) was improved by better background detection, and length, width, and height estimation were established directly from the reconstructed 3D object. First length was defined as the longest distance inside the object, then width and height were defined as the longest distances along directions orthogonal to length and to each other, with width always being the longer of the two distances (ISMA Editorial Board for ®Seed Identification Guide, 2024). Furthermore, for the case of a part of the seed being hidden inside the nozzle, an extrapolation was implemented to reduce the underestimation of volume. As an additional seed trait, sphericity was defined as ratio of surface area of a sphere with seed volume and actual surface area of the seed. These changes are explained in detail in the **Supplementary Data Sheet 1**.

Seed colour information was retrieved from the images of the 2D station of the *pheno*Seeder. To characterize seed brightness, we used the V channel of a HSV colour representation. To avoid edge effects, the mean and standard deviation of a cut out of 5 by 5 pixels (corresponding to ca. 80 x 80 µm) from the centre of each image was calculated, retaining some of the variation of brightness on a seed surface without including darker pixels at the edge of the seed.

### 2.4 Sowing of many small seeds

**Figure 3:**
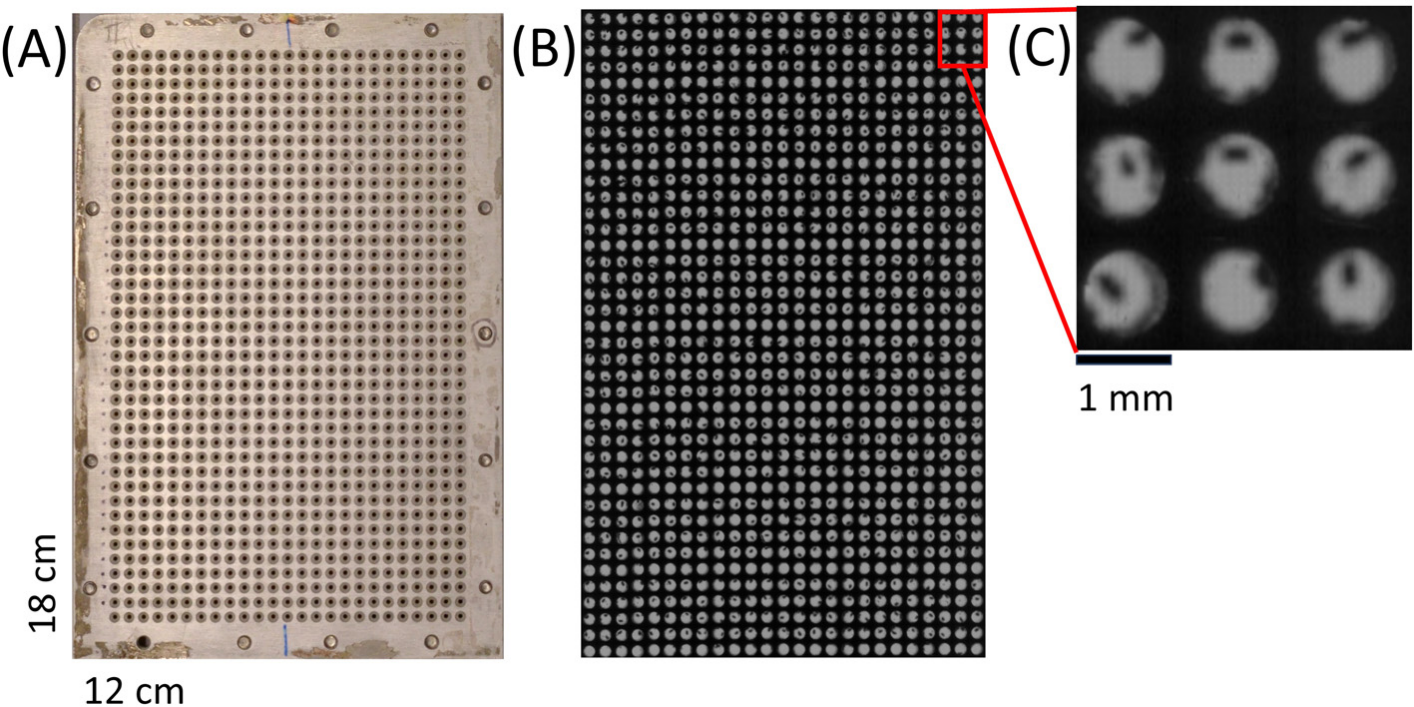
Intermediate storage for Arabidopsis seeds. **(A)** Image of custom intermediate storage for 1000 Arabidopsis seeds after phenotyping. **(B)** Combination of six scanned images of the intermediate storage for visual inspection. **(C)** 40 x magnified cut-out of the complete scanned image for validation.

A standard cycle of phenotyping one Arabidopsis seed with the *pheno*Seeder takes approx. one minute, mainly because of the time needed for image acquisition at the 3D station and for equilibration of the balance. This limits the number of seeds which can be sown on soil directly after phenotyping, because a long time interval between first and last seed sown on a tray could affect the germination time of those seeds. Thus an intermediate storage of individual seeds is important for sowing many Arabidopsis seeds and could also be used for seed classification and selection, as suggested by Jahnke et al. (2016) (Figure 5 therein). For this purpose, we used a custom-made intermediate storage consisting of two rectangular shaped plates of aluminium with dimensions 18 cm by 12 cm and a height of 1 cm. 25 x 40 holes of diameter 1 mm were drilled into each of the plates (**Figure 3A**). A steel mesh (mesh width 0.077 mm according to DIN ISO 9044) was fitted between the plates before assembly. The mesh prevents the seeds from falling through the bottom holes. The holes in the bottom are serving two purposes, ensuring a directed airflow when using negative pressure to retrieve the seeds, and providing enough transparency for visual detection. For the latter the storage plate was scanned (Epson Expression 10000 XL, Suwa, Japan) several times. Each time the plate was shifted two centimetres along the short side, to obtain full vertical views through 4–5 out of the 25 rows with 40 holes each. In total six images were taken, out of which the parts with complete views of the holes were extracted and combined. An example of the output is shown in **Figure 3B** and **C**. The time of intermediate storage can vary depending on the experimental setup and design. In the current study the seeds stayed in the intermediate storage for at most 7 days. The intermediate storage can be used to check seed quality and validity of seed trait data before sowing. This would allow selection of stored seeds for sowing according to different criteria, e.g., sowing seeds falling into specific classes of trait values. In the present study only the criterion of successfully determined seed traits was used to select seeds for sowing.

**Figure 4:**
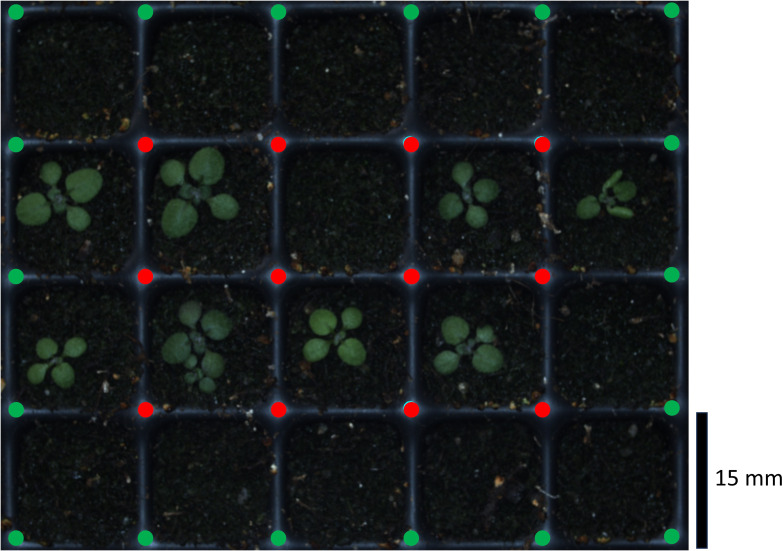
Exemplary raw image of the Growscreen with automated detection of crossings to optimize cropping of single pot images. The routine recognized the inner crossings (red points) and extrapolated the outer points (green points).

### 2.5 Improved single pot cropping

After stratification, all trays containing sown seeds were measured by the Growscreen at the same time of the day over at least 14 days. During the measurements, a camera scanned the trays by taking multiple images of 5 by 4 pots each. These images were than cropped to create one image per pot, which was linked to the corresponding seed ID (Scharr et al., 2020). So far, the cropping procedure was based on a fixed grid superimposing the images. This led to an occasional misalignment of the single pot images due to fabrication inaccuracies (up to 3 mm) and a general flexibility of the trays. In such a case, parts of a plant could be missing in the cropped image. This issue was solved by replacing the fixed grid with an algorithm which recognizes the central crossings between the pots (**Figure 4** red points). The identified points were used to draw a grid and extrapolate the outer crossing points (**Figure 4** green points). The cropping was performed along the grid lines.

### 2.6 Determination of germination time and early growth rates

**Figure 5:**
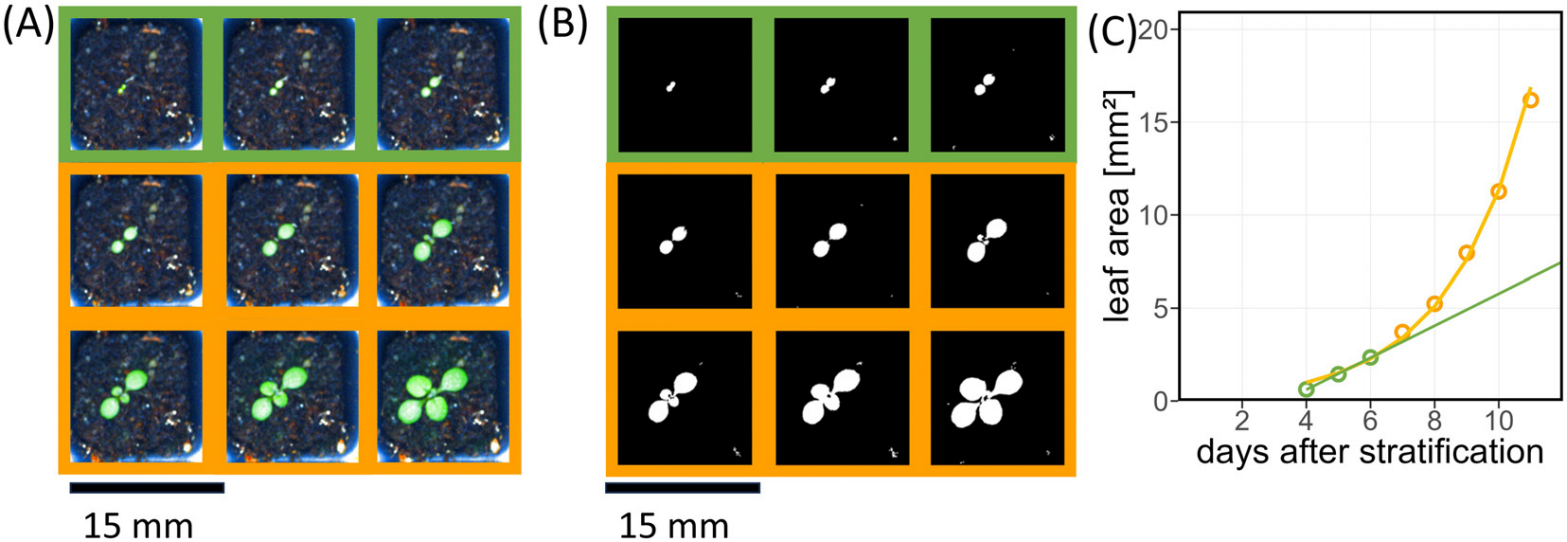
Arabidopsis plant phenotype characterized by projected leaf area during the first days after emergence. **(A)** Exemplary time series of single pot images; time points of linear growth stage are highlighted by green squares, exponential growth by yellow squares, respectively. **(B)** Same as **(A)** with segmented leaves. **(C)** Growth curve of leaf area of an example plant corresponding to **(A)**. Green line represents the linear fit based on the green points. The yellow line shows the fitted exponential growth curve based on all data points.

The single plant images (**Figure 5A**) were segmented to determine leaf area (**Figure 5B**), which was then used to quantify germination time and early leaf growth (**Figure 5C**). In Arabidopsis, growth of cotyledons is dominated by cell expansion instead of cell division (Tsukaya et al., 1994) and thus does not enter an exponential regime. Exponential increase of leaf area starts with the emergence of the first true leaf pair (Asl et al., 2011). Therefore, we approximated leaf area increase by a linear model in the first few days after emergence, followed by an exponential increase. Identification of the linear part of growth was performed backwards, i.e., by removing one data point after another from the end of the leaf area time series (**Figure 5C**), until certain criteria for a linear fit were fulfilled: adjusted R² > 0.9, both slope and intercept significantly different from 0, and deviance divided by the number of observations below 0.1. For the remaining data points (**Figures 5A and C** highlighted in green), the slope of the linear fit defined the early growth rate, and the intercept of the linear fit line with the x-axis was used to determine germination time.

### 2.7 Simulation of “old” balance for calculating a minimal number of measurements

The data obtained by the new balance for batches C and T were used to simulate the uncertainty of the old balance. Around each data point a normal distribution was built with a standard deviation of 15%, representing the repeatability precision of the old balance used by Jahnke et al. (2016). By drawing 100 values from each of these distributions a total distribution for each batch was generated. The t-value from a comparison of the two batch distributions was calculated depending on n. By comparing with tabulated t-values, a critical value of n was determined for a certain significance level.

### 2.8 Data analysis

The data was analysed with R (R Core Team, 2022) using the packages ggplot2 (Wickham, 2016), tidyverse (Wickham et al., 2019), broom (Robinson, 2014), rstatix (Kassambara, 2022) and magick (Ooms, 2021). T-tests were performed to test for significance of treatment effects. Confidence intervals of standard distributions were calculated using the single-sample chi-square test (Sheskin, 2000).

## 3 Results and discussion

### 3.1 Performance test *pheno*Seeder

**Figure 6:**
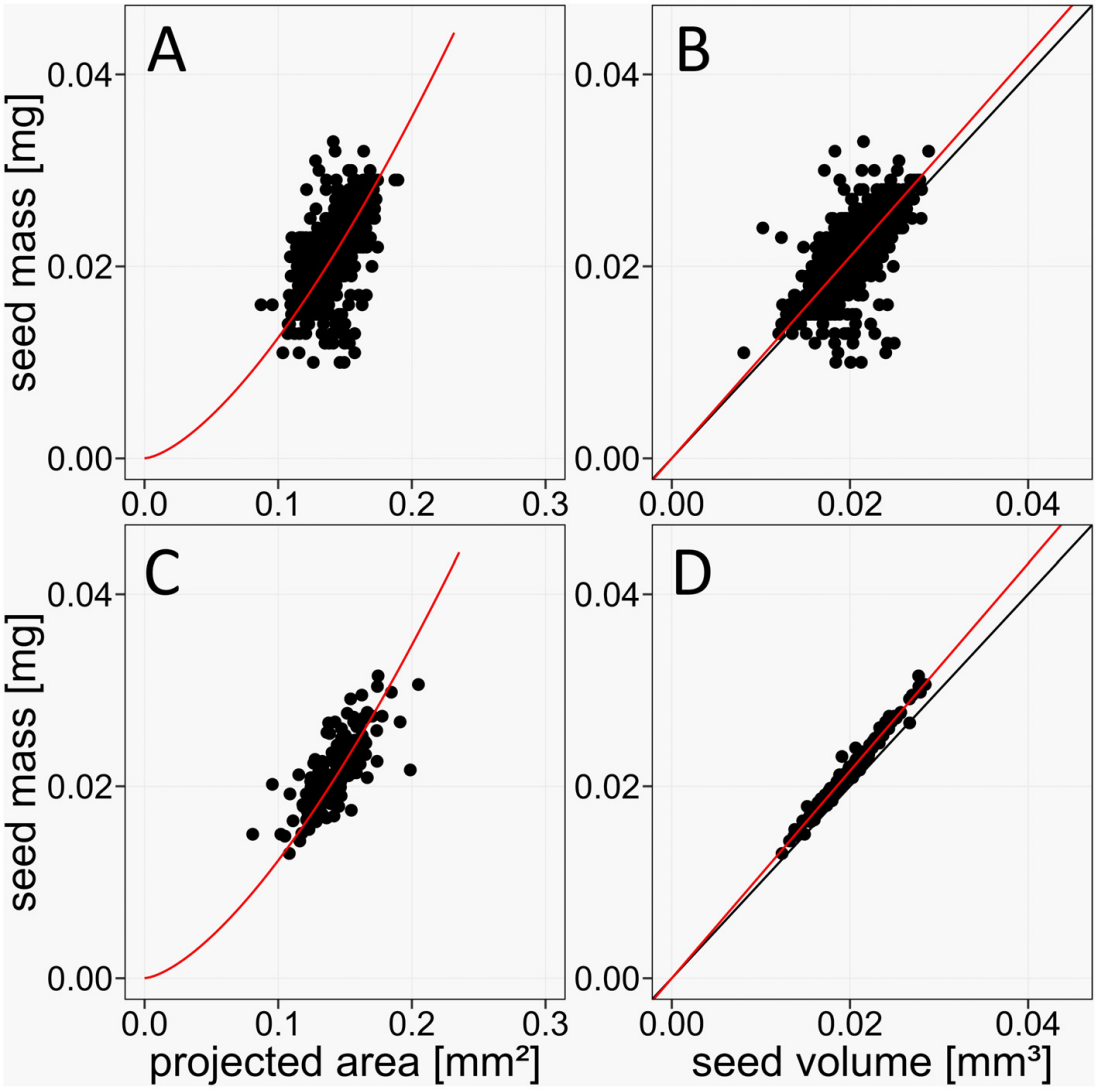
Improved accuracy of the phenoSeeder due to higher resolution of balance and changed method of volume estimation. **(A, B)** Correlations of seed mass vs. projected seed area and volume, respectively, for data of Arabidopsis Col-0 from Jahnke et al., 2016, *n* = 1006, Pearson correlations 0.59 and 0.71, respectively. **(C, D)** Correlations of seed mass vs. projected seed area and volume, respectively, in the current study, *n* = 187, Pearson correlations 0.75 and 0.98, respectively. Red lines represent the linear fit *y* = *ax* **(B, D)** and the non-linear fit *y* = *ax* ^3/2^ **(A, C)**, respectively. Black lines are identity lines.

To test the performance improvement of the *pheno*Seeder for Arabidopsis seeds by the more precise balance and the improved volume estimation method, we investigated the correlation between seed mass and volume for the combined control seed batches G0 and C. With the new equipment we achieved a Pearson correlation of 0.98 (**Figure 6D**), compared to a value of 0.71 in the published version of the *pheno*Seeder (**Figure 6B**) (Jahnke et al., 2016). From mass and volume, seed density can be calculated for each seed. The quality of the linear fit in **Figure 6D** indicates that density was independent of seed mass and volume for our data. In such a case density can be employed to validate the weight measurements and volume estimation. Any value of density above or below a certain threshold could be an indicator of an invalid mass and/or volume estimation. Deviations could occur if multiple seeds, broken seeds or any other objects such as dust and soil particles were picked up by the seed handling tool.

Jahnke et al. (2016) argued that projected seed area, commonly termed “seed size”, is not a suitable proxy for seed mass, because it is a two-dimensional quantity and missing the information about seed height. This was demonstrated for larger seeds of rape and barley, but could not be clearly shown for Arabidopsis because of the lacking precision of the balance. The Pearson correlation between projected seed area and seed mass of Arabidopsis Col-0 for the data of Jahnke et al. (2016) was 0.59 (**Figure 6A**). With the new balance the correlation of projected area and mass increased to 0.65 (**Figure 6C**), which is still not sufficient for using projected area as a proxy for mass. When approximating mass from projected area *A* by the equation *y* = *a A* ^3/2^ already used by Jahnke et al. (2016), individual deviations between actual seed mass and mass estimated from projected area can be as high as 37%.

In order to separately quantify the performance of volume estimation and seed mass determination, single seeds of Arabidopsis Col-0 were measured repeatedly multiple times (**Tables 1** and **2**). The standard deviation of seed mass resulting from this reproducibility test was 0.1 µg which is below the reproducibility of 0.25 µg specified by the balance manufacturer. It corresponds to a relative standard deviation (RSD) ranging from 0.4 to 0.5%. This is a substantial reduction of variability compared to Jahnke et al. (2016), where the RSD values for seed mass were much higher, ranging from 12 to 29% (**Table 1**). These values were obtained for the different Arabidopsis genotype Lag2-2. However, the comparison is valid because it is just about objects with similar masses and shapes.

**Table 1:** Reproducibility test of seed weighing.

Source	Species and Accession	No.	*n*	Seed mass [µg]
Jahnke et al., 2016	Arabidopsis Lag2-2	1	60	16.9 ± 2.5
		2	60	9.9 ± 2.9
		3	60	18.4 ± 2.2
This study	Arabidopsis Col-0	1	100	25.5 ± 0.1
		2	100	22.5 ± 0.1
		3	100	21.0 ± 0.1

Mean of seed mass ± standard deviation for three individual seeds measured repeatedly; *n* = number of repetitions; No. = seed number.

**Table 2:** Reproducibility test of seed volume estimation.

Seed number	Previous version	This study
1	24.2 ± 0.6	24.4 ± 0.4
2	21.0 ± 0.5	21.1 ± 0.3
3	19.0 ± 0.6	19.6 ± 0.4

Mean of seed volumes [mm³] ± standard deviation in a comparison between previous and latest version of the volume estimation method. Three different seeds of Arabidopsis Col-0 were measured *n* = 100 consecutive times.

During each measurement cycle in the *pheno*Seeder, the images for the volume estimation were acquired as well. These were utilized for estimating the seed volume (**Table 2**) with the prior version (Roussel et al., 2016) and the latest version of 3D surface reconstruction. For the updated version of volume carving, the standard deviation decreased to a range from 1.54 to 1.90%, compared to previous range from 2.45 to 3.10%. The remaining variability of volume in the repeatability measurements is mostly due to the resolution of the camera and a general limitation of the optical measurement to recognize grooves and surface irregularities, which strongly depends on the seed position at the tip of the nozzle.

While optical methods have certain limitations, we are not aware of an existing more precise method that is equally non-invasive, automated for individual seeds and with a decent throughput. Computer tomographic (CT) measurements can recognize surface features, which is essential for volume determination. Additionally, it is possible to investigate the void parts inside the seed (Karahara et al., 2015; Schmidt et al., 2020), and high resolution CT is able to measure internal structures such as the embryo or even parts of the embryo like cotyledons or hypocotyl. Those properties can be related to the germination ability and the early seedling development (Karahara et al., 2023). However, there is still an uncertainty whether germination behaviour changes for seeds analysed with x-rays, an automated measurement set up for individual seeds is still missing, despite efforts by Porsch (2020) to automate batch analysis of sugar beet seeds, and time for measuring individual seeds of Arabidopsis in high resolution is limiting throughput (Karahara et al., 2015). On the other hand, the acoustic volumeter method by Sydoruk et al. (2020) is fast and can be easily automated by integration into a weighing station of the *pheno*Seeder, but would require a new design to be suitable for the very small seeds of Arabidopsis.

### 3.2 Correlations between traits in the seed-to-plant tracking pipeline

**Figure 7:**
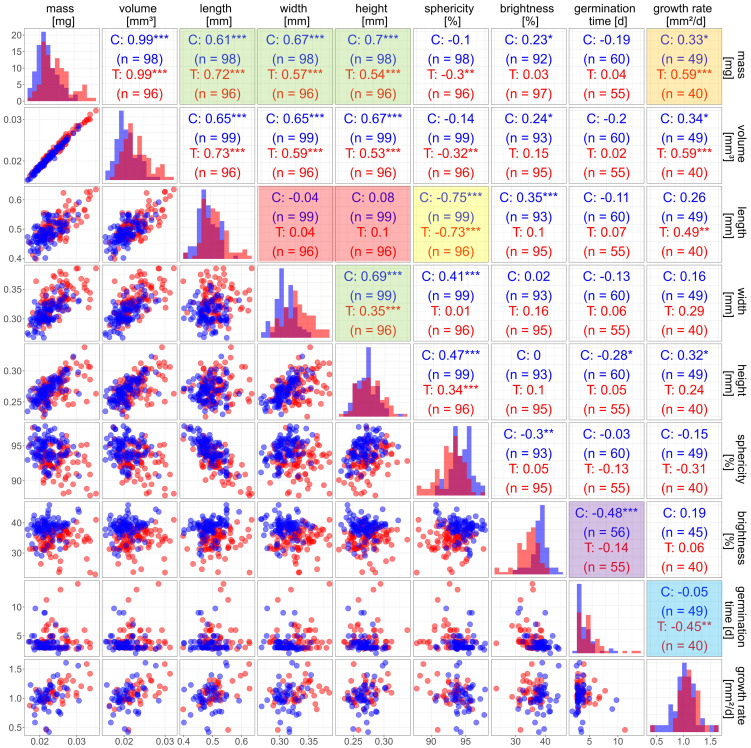
Correlation matrix for seed and plant traits of Arabidopsis Col-0 from elevated temperature (T, red) and control (C, blue). The diagonal contains density plots for each trait. The lower part contains scatter plots for each pair of traits. *n* is the number of observations. The upper part contains the Pearson correlation coefficients. Significance of correlation is denoted by * *p* > 0.05; ** *p* > 0.01; *** *p* > 0.001. Coloured tiles see text.

A main benefit of seed-to-plant tracking is the possibility to analyse correlations between all traits of seeds and corresponding plants. For the main traits obtained in the seed-to-plant tracking of batches C and T from control and elevated temperature, respectively, the correlation plot in **Figure 7** provides an overview of individual data.

There was a significant (p<0.05) difference between the means of the two groups for all traits except growth rate (**Table 3**). Seed mass and seed volume increasing by 12% and 13%, respectively, for seeds from mother plants exposed to elevated temperature. We calculated how many seeds would have to be measured to identify these differences. For the new balance it is necessary to measure at least 15 seeds for both batches to detect the differences in the distribution means to a significance level of 95%. The balance used by Jahnke et al. (2016) would require at least 28 measurements per batch. On first sight this looks like an impressive improvement and a very low number. However, in addition to the distribution mean, we also want to precisely estimate the standard deviation of the distribution, which requires a much larger number of seeds to be measured. The 95% confidence intervals of the estimated standard distribution *σ* range from 41% of *σ* for 50 seeds, to 28% for 100 seeds and 20% for 200 seeds. Thus, increasing the number of measured seeds from 100 to 200 provides only little improvement, so we consider 100 seeds as a good compromise between a highly precise estimation of the distribution mean and a sufficiently precise characterization of the standard deviation.

**Table 3:** Summary for all statistical tests performed.

Trait	Mean_C_	Mean_T_	SD_C_	SD_T_	*n*_C_	*n*_T_	*p*
seed mass [µg]	22.1	24.8	2.92	3.95	98	98	1.6E-07
seed volume [nl]	20.5	23.0	2.69	3.46	99	96	3.5E-08
3D length [mm]	0.49	0.51	0.04	0.05	99	96	5.1E-05
3D width [mm]	0.31	0.33	0.02	0.02	99	96	1.4E-11
3D height [mm]	0.27	0.27	0.02	0.02	99	96	0.028
sphericity [%]	94.5	92.9	1.64	1.95	99	96	4.4E-09
seed brightness [%]	38.4	34.2	3.01	3.66	94	97	2.1E-15
germination time [d]	3.77	4.62	1.50	2.33	60	56	0.022
growth rate [mm²/d]	1.02	1.09	0.23	0.19	49	40	0.093

SD is standard deviation, subscripts C and T denote seeds from control and elevated temperature, respectively. *n*, number of measurements. *p* values were obtained from a two-sided t-test.

The correlation between seed mass and volume was equally high for the treated seeds as for the control (**Figure 6D**). No differences in seed density could be observed between the two batches. Therefore, it is not surprising that correlations of other traits with volume and mass differed only marginally (**Figure 7**). Thus, in the following we will only use seed mass for discussion of correlations.

There was a strong correlation between mass and length, width, and height of the seeds (**Figure 7**, upper green box) but not as strong as between mass and volume. This is due to each one-dimensional trait (length, width and height) only covering part of the three-dimensional extension of a seed. Seed width correlated significantly with seed height (**Figure 7**, lower green box). However, neither of those two traits correlated with the seed length (**Figure 7**, red box). This indicates that a change in volume could either be dominated by a change in length or a change in width and height. This finding is supported by a negative correlation (*r* = -0.75; -0.73 for control and treatment, respectively, see **Figure 7**, yellow box) between seed length and sphericity, in contrast to no correlation between seed mass and sphericity, implying possible subgroups of seeds with different developmental characteristics. The reasons are not clear. Options would be intra- or inter-plant differences in, e.g. mechanical constraints (see Rolletschek et al., 2024 for the case of rape seeds), or different seed development depending on age of the mother plant (Dogra and Dani, 2019).

The mathematical procedure used to identify seed length, width and height is purely based on distances in orthogonal directions in a three-dimensional object and does not consider the characteristic shape of Arabidopsis seeds. Therefore, in some cases width and height could be interchangeable. However, this uncertainty does not play a role for the correlations discussed above, because width and height have almost the same range and show similar correlations.

We used seed brightness as an optical trait, because it is straightforward to interpret. There was a small but significant difference in mean seed brightness between batches, with a brightness of about 34% for treated seeds and a brightness of 38% for the control batch. The darker seeds could be a hint at a change in the composition of the seed coat. The brown colour of Arabidopsis seeds originates from proanthocyanidins, which oxidize and become brown during seed ripening (Lepiniec et al., 2006). There might be more proanthocyanidins synthesized during the treatment under elevated temperature, leading to darker seeds. The only relevant relation for seed brightness was the highly significant negative correlation with germination time for the seeds of the control group (**Figure 7**, purple box). The seed coat provides a certain mechanical resistance for the radical and a barrier for water and oxygen, and darker seeds seem to be more dormant than brighter seeds (Debeaujon et al., 2000). This aligns with our results indicating that brighter seeds take less time to germinate than darker seeds (**Table 3**). However, the correlation was dominated by the brighter seeds, which were mostly present in the control batch. Therefore, it is difficult to draw a general conclusion from our data. The other values of the HSV colour representation were not included in the correlation analysis, because they did not add further insight: saturation showed only little variation inside and between batches, whereas hue correlated strongly with brightness. The data of hue and saturation are included in **Supplementary Table 4**.

There was a tendency that seeds from elevated temperature had a higher growth rate, yet a t-test did not indicate a significance (**Table 3**). We observed a one-day delay in mean germination time for the treatment group (**Table 3**). There was a tendency of a positive correlation between growth rate and seed mass in the control group. In the treatment group this correlation was significant (**Figure 7**, orange box). A higher seed mass might be advantageous by enhancing the growth and survivability during the initial phase after emergence due to more reserves or an overall bigger embryo (Krannitz et al., 1991; Alonso-Blanco et al., 1999). Interestingly we detected no correlation between seed traits and germination time. The only relation found was a moderate negative response between growth rate and germination time for seeds of the treatment (**Figure 7**, blue box).

Generally, the reliability of correlations depends on the number of valid data points. Because of the limited germination rates in natural soil, seedling numbers are the bottleneck limiting the number of data points in the correlations between seed and plant traits. Also, correlation coefficients tend to depend on extreme values. Therefore, we considered correlation coefficients as well as significances for evaluating the correlations.

### 3.3 Possible further amendments

The seed-to-plant tracking provides a pipeline for phenotyping individual seeds as well as emergence, which could be easily extended. In our study we focused on aboveground traits of very early plant development. Properties of the exponential growth phase, number of leaves, and rosette shape are already part of the automated Growscreen data acquisition (Scharr et al., 2020). Other traits could be linked to the seed ID, such as manual recording time points of developmental stages, e.g. flowering time and ripening of siliques, or additional automated measurement modalities, e.g. hyper-spectral imaging (Jansen et al., 2009). There are approaches to characterize belowground traits of Arabidopsis like root architecture (Nagel et al., 2020), but integration of such modalities into the seed-to-plant tracking will be difficult.

Despite the presented improvements of the modalities included in the seed-to-plant tracking, it is still necessary to manually validate the data, in particular the Growscreen results. The identification of plant appearance by the occurrence of at least ten green pixels is prone to some uncertainties. Therefore, it is necessary to verify the appearance of a plant if the number of pixels is near the threshold. Similarly, a validation of growth curves is needed in cases of leaves initially folded between soil particles or issues with leaf identification after segmentation. The workload of this manual validation could be reduced by training neuronal networks (Samiei et al., 2020) to identify leaves by shape. With our study, we provided an annotated data set which could be used for such training purposes.

## 4 Conclusion

In the present study we introduced the seed-to-plant tracking pipeline, which enables characterizing individual seed traits (e.g. mass, volume, etc.) as well as corresponding plant traits (germination time, growth rate) in a high precision for the model plant Arabidopsis grown on soil, which is challenging in terms of seed mass and volume as well as plant size.

The pipeline not only enables selecting seeds based on certain trait values, but also would allow selection of plants with defined growth properties for propagation. Our seed-to-plant tracking enables comprehensive phenotyping over multiple generations, thus observing and characterizing transgenerational phenotypic changes under different environmental conditions, which could be linked with genetic information elucidating mechanisms of inheritance.

## Data Availability

The datasets presented in this study can be found in online repositories. The names of the repository/repositories and accession number(s) can be found below: https://doi.org/10.26165/JUELICH-DATA/KZDQYD.

## Supplementary material

Supplementary Table 1 Data of seed mass vs volume and projected seed area, respectively, shown in **Figure 6**.

Supplementary Table 2 Data of repeatability measurements analysed in **Table 1** and **2**.

Supplementary Table 3 Data of leaf area time series used for estimation of plant growth.

Supplementary Table 4 Data of seed and plant traits used for correlation analysis in **Figure 7**.

Supplementary Data Sheet 1 Detailed description of improvements to the 3D surface reconstruction.
