# Supplementary material for: Seed-to-plant-tracking: automated phenotyping of seeds and corresponding plants of Arabidopsis
Source: Front Plant Sci. 2025 Apr 28;16:1539424. doi: 10.3389/fpls.2025.1539424 (PMC12066762; doi:10.3389/fpls.2025.1539424)
Supplement: Supplementary Data Sheet 1 — Detailed description of improvements to the 3D surface reconstruction. [file DataSheet1.pdf]

Supplementary Material S1. Detailed description of improvements to the 3D surface reconstruction

Here we detail improvements to the 3D surface reconstruction method of Roussel et al. (2016) to achieve a more reliable, accurate and precise estimation of seed volume. The image analysis was implemented in Python, using packages SciPy (Virtanen et al. 2020, https://scipy.org/), NumPy (Harris et al. 2020, https://numpy.org/), OpenCV (Bradski 2000, http://opencv.org/) and Open3D (Zhou et al. 2018, https://www.open3d.org/).

Separation of seed from background

Roussel et al. (2016) applied a fixed threshold to separate pixels of the seed from the image background. This method has the disadvantage of introducing inaccuracies when seeds in a batch have different luminance. Due to a limitation of the optical system, there will always be a diffuse transition of brightness at the edge between the seed area and the background, and for a higher difference between (dark) seed and (bright) background a fixed absolute threshold will lead to an overestimation of the number of pixels counted to the seed. This is especially relevant for objects as small as Arabidopsis seeds with an average diameter of only ca. 40 pixels on the 2D image. Here an uncertainty of the seed separation of just one pixel in each direction already leads to a relative volume error of ca. 4%. Therefore, we replaced the threshold method by an edge detection method without any fine-tuning parameters such as a fixed absolute threshold, which is universally applicable for seeds of different sizes and colors. This method to separate the seed and nozzle from the background consists of four steps:

- Creating a coarse background mask. First, for all pixels a distribution of brightness variance is determined over all single projections obtained during the nozzle rotation. Noting that these distributions are dominated by a maximum at low variance from the background and another maximum at high variance from the seed and nozzle positions, we calculate the minima of the variance distributions as proxy for the transition regions between background and seed with nozzle. Then, background masks are calculated for each single projection using the minima and a FloodFill method of the OpenCV package starting from a corner of the images.
- Creating a mask of edge locations, i.e., the seed-to-background border locations, which are local maxima of the derivative images. This is achieved by applying filters with 18 rotations of a half 2D derivative Gaussian kernel (Magnier et al. 2019 and 2023). This method is preferential to other available methods, e.g., multidimensional gradient magnitude using Gaussian derivatives (scipy.ndimage.gaussian_gradient_magnitude), which tend to generate holes in the mask (red circles in Supplementary Figure 1B). Such holes can lead to a “penetration” of the background pixels into the seed/nozzle related pixels during the following step. The method by Magnier et al. (2019 and 2023) avoids this by retrieving more pixels related to the edges, especially at places where two edges are crossing (Supplementary Figure 1C).
- Creating a fine background mask (Supplementary Figure 2) by recursively adding those pixels to the borders of the coarse background mask which are not laying on the edges calculated before.
- Applying an opening morphological transformation to the obtained mask (OpenCV method morphologyEx with cv.MORPH_OPEN as type of morphological operation) to remove individual pixels outside of seed and nozzle.

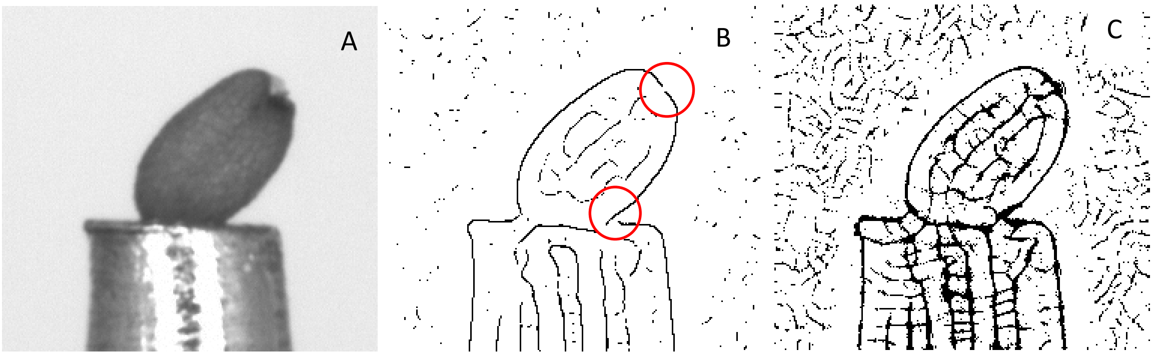

Supplementary Figure 1. Comparison of masks of the seed-to-background border locations from the original image (A) created either by multidimensional gradient magnitude using Gaussian derivatives (B) or by our method with a half 2D derivative Gaussian kernel (C). Red circles depict locations with holes in the mask.

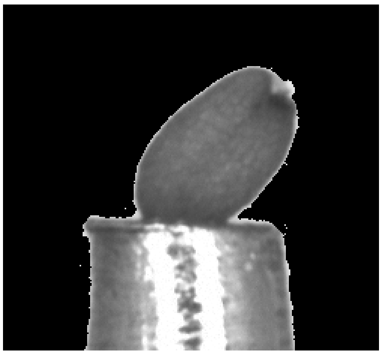

Supplementary Figure 2. Resulting separation of the seed and nozzle from the background by removing the pixels related to the fine background mask.

Extrapolation of the hidden part

Small seeds like Arabidopsis are always partly hidden in the nozzle (inner diameter of 0.15 mm). The extent of the hidden part depends on the position of the seed on the nozzle. Generally, the hidden part leads to an incomplete 3D reconstruction of the seeds and to a possible underestimation of their volume. To overcome this issue, we introduced an extrapolation of the hidden part by fitting a set of ellipses to selected voxels located on parallel planes that are orthogonal to the nozzle tip plane. Only voxels located close to the missing part of the seed were considered, and only those which are located on the seed surface and not further than 20 voxels out of the missed part. Next, the fitting procedure was repeated on other parallel planes orthogonal to the previous planes taking into account the previously extrapolated voxels. This fitting by switching the planes' directions was repeated several times leading to a smooth surface. This extrapolation is mimicking the end of seed better than an ellipsoid, which in many cases causes an unreliable solution because of the form of the seed.

Surface reconstruction

Reconstruction of a 3D surface from the data was realized using the Open3D package. First, internal voxels in the point cloud of voxels were removed by evaluating the number of the nearest neighbours. Next, a triangle mesh was computed from the remaining voxels by (1) estimating normals using the *estimate_normals* function and (2) orienting the normals using *orient_normals_consistent_tangent_plane*. Then the surface reconstruction was performed with the *create_from_point_cloud_poisson* method of the *open3d.geometry.TriangleMesh* class. If the surface

was watertight, the class methods *get_volume()* and *get_surface_area()* were used to estimate the seed volume and surface area, respectively.

References

Bradski, G. (2000). The OpenCV Library. *Dr. Dobb's Journal of Software Tools*.

Harris, C.R., Millman, K.J., van der Walt, S.J., Gommers R., Virtanan P., Cournapeau D., et al. (2020). Array Programming with NumPy. *Nature* 585, 357–362. DOI: 10.1038/s41586-020-2649-2.

Magnier, B., Moradi, B., Carré, P. (2019). Evaluation of Half Gaussian Filter Rotation for Edge Detection. *2019 8th European Workshop on Visual Information Processing (EUVIP)*, Roma, Italy, pp.52-57. DOI: 10.1109/EUVIP47703.2019.8946144

Magnier, B., Hayat, K. (2023). Revisiting Mehrotra and Nichani's Corner Detection Method for Improvement with Truncated Anisotropic Gaussian Filtering, *Sensors*, 23, 8653. DOI: 10.3390/s23208653

Roussel, J., Geiger, F., Fischbach, A., Jahnke, S., Scharr, H. (2016). 3D Surface Reconstruction of Plant Seeds by Volume Carving: Performance and Accuracies. *Frontiers in plant science* 7, S. 745. DOI: 10.3389/fpls.2016.00745.

Virtanen, P., Gommers, R., Oliphant, T.E., Haberlan, M., Reddy, T., Cournapeau, D., et al. (2020). SciPy 1.0: Fundamental Algorithms for Scientific Computing in Python. *Nature Methods*, 17(3), 261-272. DOI: 10.1038/s41592-019-0686-2.

Zhou, Q.-Y., Park, J., Koltun, V. (2018). Open3D: A Modern Library for 3D Data Processing, arXiv:1801.09847. DOI: 10.48550/arXiv.1801.09847
